# First insights into the current practice, knowledge, and attitudes of community pharmacists regarding sexual adverse drug reactions: a cross-sectional survey

**DOI:** 10.1093/sexmed/qfac014

**Published:** 2023-01-12

**Authors:** Rineke Gordijn, Melianthe P J Nicolai, Henk W Elzevier, Henk-Jan Guchelaar, Martina Teichert

**Affiliations:** Department of Clinical Pharmacy and Toxicology, Leiden University Medical Center, Leiden, 2300 RC, the Netherlands; Department of Urology, Netherlands Cancer Institute–Antoni van Leeuwenhoek Hospital, Amsterdam, 1066 CX, the Netherlands; Department of Urology and Medical Decision Making, Leiden University Medical Center, Leiden, 2300 RC, the Netherlands; Department of Clinical Pharmacy and Toxicology, Leiden University Medical Center, Leiden, 2300 RC, the Netherlands; Department of Clinical Pharmacy and Toxicology, Leiden University Medical Center, Leiden, 2300 RC, the Netherlands

**Keywords:** sexual adverse drug reactions, sexual dysfunction, pharmaceutical care–pharmacist–patient relation, communication

## Abstract

**Introduction:**

Sexual function can be negatively influenced by adverse drug reactions (ADRs) potentially caused by >300 drugs. These sexual ADRs (sADRs) can lead to low adherence and decreased quality of life. Physicians are known to barely discuss sexual function. Pharmacists also have an important role in informing and advising patients on ADRs, but it is unknown how community pharmacists deal with sADRs.

**Aims:**

The purpose of this study was to evaluate the current practice, attitudes, and knowledge of community pharmacists about informing, detecting, and discussing sADRs.

**Methods:**

An online survey with 31 questions was sent to all 1932 pharmacy members of the Royal Dutch Pharmacists Association. The survey was modified from previous surveys that questioned different medical disciplines on their practice, attitudes, and knowledge of sexual function related to their fields. Questions were added on pharmacists’ practice concerning ADRs in general.

**Results:**

A total of 97 (5%) pharmacists responded. During first dispenses of drugs, 64 (66%) informed patients on a selection of common ADRs. Almost all (n = 93, 97%) discussed diarrhea or constipation in at least half of the related occasions, whereas 26 to 31 (27%-33%) discussed sADRs. The sADRs for high-risk drugs were more often named at first than at second dispenses (n = 61 [71%] vs n = 28 [32%]). Pharmacy technicians were generally considered not to discuss sADRs (n = 73, 76%; never or in less than half of the occasions). Lack of privacy (n = 54, 57%) and language barriers (n = 45, 47%) were the most acknowledged barriers to discuss sADRs. Moreover, 46% (n = 45) considered their knowledge insufficient to discuss sADRs. Responsibility for informing, advising, and detecting sADRs was most often attributed to pharmacy technicians (n = 59, 62%), pharmacists (n = 46, 48%), and patients (n = 75, 80%), respectively.

**Conclusion:**

This study shows that one-third of pharmacists and two-thirds of pharmacy technicians barely talked about sADRs during first dispenses for high-risk drugs. The low response rate suggests that mostly interested pharmacists responded, thus likely overestimating the sADR discussion rate. To provide patients with unique opportunities to discuss sADRs in community pharmacies, more attention is needed for raising awareness about the topic among pharmacists and for barriers such as the presence of other clients and limited knowledge about sADRs.

## Introduction

One in 11 inhabitants of the Netherlands is prescribed ≥1 drugs with high risks for sexual adverse drug reactions (sADRs).^1^ Examples of common sADRs are decreased desire with selective serotonin reuptake inhibitors, ejaculation disorders with alpha blockers and 5-alpha-reductase inhibitors, and increased prolactin levels with antipsychotic drugs.^1^ When sADRs occur, patients’ quality of life, relationships, and drug adherence may be negatively influenced.^2^ Although many patients want to discuss sexual function or sADRs with their health care providers,^3^^,^^4^ medical doctors and nurses rarely provide opportunities to talk about this important part of their patients’ lives.^5-7^ Concerning sADRs, this may also be discussed with pharmacists, since informing and advising about adverse drug reactions (ADRs) is part of a pharmacist’s daily practice and responsibilities. However, little is known about the frequency and attitudes toward talking about sADRs in the community pharmacy.

In comparison with other health care professionals, community pharmacists have a unique context to accommodate patients who wish to discuss sexuality in a health care setting. The pharmacist is the most accessible health care professional, present in most neighborhoods and available to patients without appointment. Consequently, if patients are aware of the possibility to ask the pharmacist about sADRs, their barriers to address them or to ask other questions concerning sexual dysfunction could be diminished.

Because of the high number of drugs with potential sADRs, it is crucial to optimize pharmacies’ unique position in discussing these side effects and to inform patients adequately. Therefore, this study’s purpose was to explore the current practice, attitudes, and knowledge of community pharmacists about discussing sADRs.

## Methods

### Study design

An online survey was utilized to question community pharmacists in the Netherlands about their current practice and perspectives concerning sADRs. The survey was modified from previous surveys that questioned different medical disciplines on the practice, attitudes, and knowledge of sexual function related to their fields. With respondents’ informed consent to use their answers anonymously, the study was not subject to the Medical Research Involving Human Subjects Act per the Medical Ethical Assessment Committee of the Leiden University Medical Center (N21.063).

### Questionnaire development

The questionnaire for previous research about sexuality in health care in the Netherlands^5-7^ was modified for the tasks of community pharmacists by substituting sexual function with sADRs and by including questions on how pharmacists informed patients about ADRs in general. Questions were added on the pharmacist’s view concerning the discussion of sADRs by pharmacy technicians and the responsibility for dealing with sADRs within the multidisciplinary health care setting. The finalized questionnaire consisted of 31 questions, of which 7 were demographic questions. Most responses were scored on a 5-point Likert scale. The questionnaire was piloted by 3 pharmacists; after which, some readability adjustments were made.

### Data collection

The online questionnaire was sent to 1 email address per pharmacy for 1932 Dutch community pharmacies (96.4% of community pharmacies in 2021). The email addresses for the pharmacy members at the Royal Dutch Pharmacists Association were available; therefore, the online questionnaire was sent by this association. Its distribution method is regularly used for policy issues, generally reaching a sufficient response (~30%). The pharmacists were invited to fill in the questionnaire on September 24, 2021. A reminder was sent after 1 month. Data were collected anonymously. Questionnaires completed at least 25% were included for analysis.

### Data analysis

Descriptive statistics was performed with SPSS Statistics (version 26.0; IBM Corporation). The results that were scored on Likert scales were summarized in 2 groups: (1) never or in less than half of the occasions and (2) in at least half of the occasions.

## Results

Of the 1932 online questionnaires sent out, 97 (5%) were returned and could be included for analysis. About half the respondents were female (n = 54, 56%). The majority had worked as a pharmacist for >10 years (n = 64, 66%), spent 1 to 2 hours a day on patient contact (n = 65, 67%), and had at least 1 experience in discussing sADRs with a patient (n = 88, 91%).

### Current practice regarding ADRs and sADRs

During the first dispense of a drug, 64 respondents (66%) informed about a selection of ADRs, and 26 (27%) listed all common ADRs. During the second dispense, pharmacists more often routinely asked patients an open question regarding whether they had experienced ADRs in general (n = 69, 71%), as opposed to soliciting about specific ADRs (n = 21, 22%). Table 1 shows that diarrhea and constipation were the most common ADRs discussed for relevant drugs (n = 93, 97%; ie, in at least half of the related occasions). Regularly informing about sADRs occurred the least often: erectile dysfunction (n = 31, 33%), less desire for sex (n = 28, 30%), and vaginal dryness (n = 26, 27%).

**Table 1:** Number of pharmacists who discuss general and sexual side effects for certain situations and medications in at least in half the occasions.

	Pharmacists, No. (%)	
	Respondents	≥50% occasions^a^
“Below are different side effects listed that can be very common for a drug. How often do you discuss the very common side effect during patient consultations about a relevant drug?”		
Diarrhea or constipation	96	93 (96.9)
Different color of urine	93	86 (92.5)
Skin rash	93	84 (90.3)
Fatigue	94	84 (89.4)
Loss of taste or smell	93	69 (74.2)
Weight gain	94	60 (63.8)
Extreme sweating	92	55 (59.8)
Loss of hair	94	43 (45.7)
Vaginal infections	95	41 (43.2)
Erectile dysfunction	95	31 (32.6)
Less desire for sex	95	28 (29.5)
Vaginal dryness	95	26 (27.4)
“How often do you discuss a sexual side effect of medication with a patient during the following situations?”^b^		
First dispense of a drug with high risk for sexual adverse drug reactions (according to drug label)	86	61 (70.9)
Medication review	87	56 (64.4)
Conversation about a drug of which a potential influence on sexual function is derivable from the working mechanism (eg, influencing sexual reproductive organs)	87	53 (60.9)
Conversation in consultation room about chronic medication	87	46 (52.9)
Conversation via telephone/video about chronic medication	86	41 (47.7)
Conversation in which the context of the patient is discussed (daily activities, home situation, etc)	87	32 (36.8)
Second dispense of a drug with high risk for sexual adverse drug reactions (according to drug label)	88	28 (31.8)
“How often do you discuss the risk for sexual side effects when you inform patients with the following medication about side effects?”^b^		
Antidepressants (eg, sertraline)	87	56 (64.4)
5-alpha-reductase inhibitors (eg, dutasteride, finasteride)	88	42 (47.7)
Antipsychotics	87	40 (46.0)
Alpha blockers (eg, tamsulosin, silodosin)	87	34 (39.1)
Beta blockers (eg, metoprolol)	88	25 (28.4)
Hormonal contraceptives	88	21 (23.9)
Metformin	87	17 (19.5)
Calcium antagonists (eg, amlodipine)	88	14 (15.9)

a Respondents who chose the options “in half of the occasions,” “in more than half of the occasions,” and “always or almost always.”

b These questions were not asked to respondents who had never discussed sexual adverse drug reactions with patients (n = 9).

During the first dispense of drugs with a high risk for sADRs, 61 (71%) of 86 responding pharmacists discussed sADRs in at least half the occasions and 25 (29%) never or in less than half the occasions. Table 1 shows that the patient conversations about sADRs most frequently involved antidepressants. Pharmacy technicians were perceived by 73 (76%) of 96 responding pharmacists to never discuss sADRs or to do so in less than half the occasions.

### Views on talking about sADRs

Most respondents considered discussing sADRs important (n = 63, 65%) or very important (n = 16, 17%). The majority assigned the responsibility for detecting sADRs to patients (n = 75, 80%) or their partners (n = 73, 78%). The pharmacy technician was most often chosen as having a role in informing about potential sADRs (n = 59, 62%), whereas advising about sADRs was most commonly attributed to the pharmacists themselves (n = 46, 48%). Most respondents appointed the main responsibility for detecting and discussing sADRs to the prescriber in general (n = 45, 46%), explicitly to the general practitioner (n = 18, 19%), and less to the pharmacist (n = 13, 13%) and the patient (n = 12, 12%).

**Figure 1:**
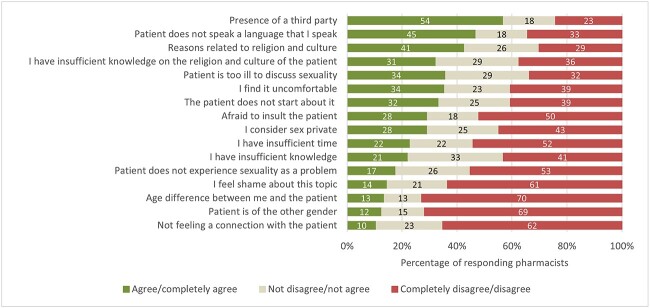
Pharmacists’ agreement with reasons to not inform about sexual adverse drug reactions. The bars represent the percentage of pharmacists with each response; the numbers inside the bars indicate how many pharmacists chose the answer option.

Figure 1 shows that the most common barrier to inform about sADRs was the presence of a third party (and thus a lack of privacy), agreed on by 54 (57%) respondents. Other barriers routinely recognized were language (n = 45, 47%), patient being too ill to discuss sexuality (n = 34, 36%), and being uncomfortable with the question (n = 34, 35%). Reasons related to religion and culture were also frequently recognized (n = 41, 43%), explained by insufficient knowledge about the patient’s religion and culture for 31 of the 41 pharmacists.

### Knowledge and skills regarding sADRs

The respondents considered themselves more often skilled to discuss sADRs (n = 76; 79%) compared to pharmacy technicians (n = 48; 50%). Forty-five respondents (46%) considered their knowledge about sADRs insufficient to discuss them. The available material to inform and advise about sADRs in the pharmacy was regarded sufficient by 53 pharmacists (55.8%). Sixty-one respondents (63%) believed that training would help them to inform about and discuss sADRs. Other regularly chosen support tools were websites for information about sexual problems (n = 45, 47%), videos about sADRs (n = 47, 49%), and patient information folders about sexuality (n = 35, 41%).

## Discussion

This study is the first to explore pharmacists’ current practice and attitudes regarding sADRs. The low response rate suggests that most pharmacists considered the topic uncomfortable, not interesting, or not a priority. Even among those who responded, about one-third never or barely discuss sADRs during first dispenses of high-risk drugs, and two-thirds reported that their pharmacy technicians never or barely discussed the topic. Nevertheless, the majority of respondents considered conversations about sADRs important and believed the pharmacy team to be responsible for informing and advising about sADRs. The perceived shared responsibility with the prescriber is also important to note because if prescribers and pharmacists do not align the sADR information that they share with the patient, this will increase patients’ worries and anxiety about medication usage.^8^

In comparison with other health care providers, pharmacists in this study experienced different barriers to discuss sADRs. The most important one, the presence of a third party, can be explained by the fact that drug dispenses traditionally take place at the counter, impeding a private consultation when other clients are nearby. Pharmacists also felt less hindered by a lack of time than other health care professionals. In addition, they agreed more often with the barriers that sex is private, that talking about sex is uncomfortable, and that they are afraid to insult the patient.^6^^,^^7^ Although pharmacists and prescribers both have the duty to provide patients with drug information, the barriers recognized by pharmacists reflect a different relationship with patients in comparison with other health care professionals.

Assets of this study include a focus on a health care professional that has been sparsely researched in the field of sexual medicine and a nationwide study population. Limitations include the cross-sectional design, self-reported frequencies of discussing sADRs, and, most important, the low number of respondents, which suggests potential nonresponse bias and limits the generalizability of the findings. Possible reasons for the low response rate are the loaded subject, the length of the questionnaire, email invitations that could have addressed persons who felt ineligible to answer (eg, manager, pharmacy technician), and survey fatigue. Concerning the length, questionnaires with similar lengths but on paper and with 1 more reminder were sent to other health care professionals in the Netherlands and received significantly higher responses (54%-64%).^5-7^ It is therefore more likely that pharmacists did not consider the topic relevant. Indeed, Dutch community pharmacists responded infrequently to a survey with a similar method on the unprioritized topic “research participation” (response rate, 9%).^9^ This study’s response therefore suggests that the findings be viewed as the opinion of the few pioneers who considered the topic important and that future pharmacist questionnaires about unpopular topics take place in more personal contexts (eg, at a congress).

These findings cannot be interpreted without considering the relative novelty of pharmaceutical care. The transition during the last decades from product- to patient-focused activities has regularly been hindered by barriers such as lack of reimbursement, lack of competency, and lack of support and relationships with general practitioners.^10^ In that respect, it may be not surprising that pharmacists in this study expressed different barriers than other health care professionals. In comparison with the high number of barriers, only 2 facilitators could be extracted from this study: an accessible private area and training for pharmacy teams. Furthermore, the study’s low response highlights that this topic requires more attention among pharmacists before interventions toward patients can be designed. Because of the high number of drugs with potential sADRs and the potential negative consequence for patients, more attention is needed for discussions between pharmacists and prescribers about how to provide sADR care, taking into account the facilitators and barriers presented in this study.

## Data Availability

The data generated and analyzed during the current study are available from the corresponding author on reasonable request.
